# Multimodal Biometric Recognition Based on Convolutional Neural Network by the Fusion of Finger-Vein and Finger Shape Using Near-Infrared (NIR) Camera Sensor

**DOI:** 10.3390/s18072296

**Published:** 2018-07-15

**Authors:** Wan Kim, Jong Min Song, Kang Ryoung Park

**Affiliations:** Division of Electronics and Electrical Engineering, Dongguk University, Seoul 100-715, Korea; daiz0128@naver.com (W.K.); whdwhd93@gmail.com (J.M.S.)

**Keywords:** biometrics, finger-vein, finger shape, CNN, multimodal biometrics

## Abstract

Finger-vein recognition, which is one of the conventional biometrics, hinders fake attacks, is cheaper, and it features a higher level of user-convenience than other biometrics because it uses miniaturized devices. However, the recognition performance of finger-vein recognition methods may decrease due to a variety of factors, such as image misalignment that is caused by finger position changes during image acquisition or illumination variation caused by non-uniform near-infrared (NIR) light. To solve such problems, multimodal biometric systems that are able to simultaneously recognize both finger-veins and fingerprints have been researched. However, because the image-acquisition positions for finger-veins and fingerprints are different, not to mention that finger-vein images must be acquired in NIR light environments and fingerprints in visible light environments, either two sensors must be used, or the size of the image acquisition device must be enlarged. Hence, there are multimodal biometrics based on finger-veins and finger shapes. However, such methods recognize individuals that are based on handcrafted features, which present certain limitations in terms of performance improvement. To solve these problems, finger-vein and finger shape multimodal biometrics using near-infrared (NIR) light camera sensor based on a deep convolutional neural network (CNN) are proposed in this research. Experimental results obtained using two types of open databases, the Shandong University homologous multi-modal traits (SDUMLA-HMT) and the Hong Kong Polytechnic University Finger Image Database (version 1), revealed that the proposed method in the present study features superior performance to the conventional methods.

## 1. Introduction

Biometrics are methods used for user identification using the unique behavioral and physiological elements of the individual, including, inter alia, face, fingerprint, iris, and vein recognition. These biometrics are used in a variety of fields, such as system security, electronic payment, patient management in hospitals, access control, etc. Moreover, recently, the smartphone popularization has led to the emergence of biometric authentication technologies, even other than the personal computer (PC) environment. Because it features not only easier acceptability, but also a higher security level and recognition accuracy than other conventional methods, such as passwords, patterns, and personal identification numbers (PINs), which are more likely to be forged or lost, biometrics have come into the spotlight in the mobile environment. Generally, after using sensors to collect and store data, biometric systems go through a preprocessing process to reconstruct the distinctive features of the original data. This preprocessing process includes region-of-interest (ROI) segmentation, feature extraction, and image enhancement. Finally, each individual is identified after selecting representative models from feature samples of each class and comparing the similarities through a quantitative measurement before making the final decision. Among these biometrics, several studies have been conducted in the past on finger-based recognition due to its various advantages. Some of these studies have proposed fingerprint recognition methods, finger-vein recognition methods that use vein-pattern features, finger shape recognition methods that are based on the geometric features of fingers, and finger-wrinkle recognition methods based on the contour of the outer surface of finger joints. Such finger-based biometrics have not only lower risks of feature loss, but also reduced costs because image acquisition is performed using smaller (miniaturized) devices than those that are used in other biometrics. However, methods that use unimodal recognition still experience difficulties in extracting patterns accurately for various reasons, such as illumination variations, finger positional variation, shading, misalignment, and quality changes caused by finger pressure with respect to the sensor, thus leading to decreased recognition performance. To overcome these limitations, the interest in multimodal biometric recognition using two or more types of biometrics combined is emerging. In relation to such a trend, in this research, finger-vein and finger shape multimodal biometrics based on a deep convolutional neural network (CNN) are proposed. Section 2 explains various conventional finger-based recognition methods.

## 2. Related Works

There are several types of finger-based biometrics, e.g., finger-vein recognition using vascular patterns under the skin, fingerprint recognition using fine wrinkled skin textures, such as ridges at the fingertips, finger shape recognition using geometric features, such as the thickness or shape of the fingers, and finger knuckle-print recognition using patterns made based on the contour (line, wrinkles, etc.) of the outer surface of the finger joints.

Particularly, most active studies have been conducted on fingerprint recognition [1,2]. Moreover, in finger knuckle-print recognition studies conducted in the past, various studies were conducted on feature extraction analyzing mostly outer surface wrinkles. Liu et al. performed finger separation in a hand image with several fingers by detecting horizontal lines. Then, he enhanced the middle and ring fingers, which have a high degree of reliability, using Gabor filtering, and finally used derivative line detection to extract the knuckle wrinkle lines [3].

Kumar et al. could decrease the impact of hand movements by extracting contour boundaries from images of the back side of fingers and detecting the area of each finger according to valley points to segment subspaces. Then, finger knuckle ROIs were configured centered on areas with many low-brightness pixels. Finally, the matching scores that were obtained by applying principal component analysis (PCA), linear discriminant analysis (LDA), and independent component analysis (ICA) to these ROI images were fused to conduct recognition [4]. Zhang et al. extracted local direction information using Gabor filters as local operators. The extracted local features were calculated inside local patches and used to express detailed features within specific regions. At this point, enlarging the Gabor filter size enables it to contain more and more global information to the point that Fourier transform coefficients can be obtained to analyze the image’s total frequency instead of extracting local information. The obtained global and local information were appropriately combined to conduct recognition [5]. In [6], two types of coding were combined to work as a band-pass filter. Then, a primary Riesz transform-based mono-signal was used for the structural analysis of edges, lines, etc. Secondary Riesz transform-based signals were used to analyze structures, such as corners and junctions, the response of image patterns. This texture-based feature-extraction method is appropriate for images containing abundant and valid line structures, and, in addition, requires relatively shorter processing times. However, in the case of people with certain skin conditions, feature extraction might be difficult while using image processing. To solve this problem, a study was conducted on finger knuckle-print matching methods [7,8,9,10]. Aoyama et al. conducted a band-limited phase-only correlation (BLPOC)-based local block matching that features higher correlation as becoming more similar images by extracting elliptic frequency bands that show the ridges of finger knuckle prints through the cross-phase spectrum calculations in phase-only correlation (POC) functions [9]. In [10], it was confirmed that the finger knuckle-print patterns of metacarpophalangeal (MCP) joints could be recognized while using BLPOC as well.

Moreover, several studies have been conducted on finger-vein recognition methods that analyze vein patterns hidden under the skin. Qin et al. used a Radon transform-based method that detects valleys using square window patches of various sizes in the vein pixels and neighboring background pixels and extracts lines corresponding to pixels that are darker than the surroundings as vein areas. This method was able not only to detect disconnected vein areas using the Radon transform, but also to extract vein lines from partial noise and irregular shading and backgrounds by inhibiting high-frequency noises [11]. Song et al. extracted uneven vein lines in all directions through the mean curvature value using geometrical brightness properties at each point of the image, and conducted matching using a binary image-based template-matching method [12]. Miura et al. was able to extract vein areas based on the fact that, if the vein profile’s curvature is large, the center position of veins can be obtained by calculating the maximum curvature of the convex areas from the cross-profile of veins. That is to say, to obtain vein patterns in various different directions from the full image, the profiles of all orientations were analyzed in order to extract vein areas by asserting that areas with higher vein center position scores were more likely to be veins [13]. Yang et al. proposed a method to extract the vein vector fields (VVF) of finger-veins based on spatial curve filters (SCF), Gaussian weight models, and curve-length fields (CLF), and conducted the matching based on phase-only correlation (POC) [14].

Miura et al. represented parts that look dark with valleys in the image due to intravenous hemoglobin’s absorption of infrared light as cross-section brightness profiles. Here, the frequency of detection of pixels was confirmed by tracking finger-vein lines that are based on the cross-sectional brightness profile of any center pixel and repeating the same process. Then, pixel areas with frequent high values were assumed as veins and were extracted to the finger-vein patterns, and areas with low values were recognized as noise [15]. Huang et al. assumed the cross-section of most fingers to be an ellipse and represented the curved areas as finger surfaces by projecting the finger surfaces in a plane. Here, to obtain accurate contour information for line segmentation of the veins, a method that finds the upper and lower finger contour lines and middle points by calculating active contour models and ellipse projective coefficients was proposed [16].

To accurately detect the exact thickness of veins in different images, Liu et al. proposed a method that performs finger-vein segmentation using a modified repeated line-tracking method that selects useful starting points while using preprocessed images as input images through rough segmentation. In other words, to extract vein patterns robustly, vein lines were tracked while moving repeatedly towards nearby pixel points, giving consideration to eight directions at all of the locations that were selected in the rough segmentation [17]. In addition to these methods, methods to improve the full finger-vein image by increasing the contrast between finger-vein patterns and the background were proposed. Pham et al. improved the contrast of finger-vein images through a method that selects pixels with the lowest filtering value as the final value by applying Gabor filtering in four directions. After this, matching was conducted based on the extracted code through local binary patterns (LBP) [18]. Syarif et al. conducted finger-vein verification through Hessian enhancement and enhanced maximum curvature (EMC) with the histogram of oriented gradient (HOG) [19]. Yang et al. decreased useless background effects by cutting finger-vein ROIs from images using finger-vein image subwindows of certain sizes. In addition, to improve the visibility of finger-vein images, the dehazing technique was used by giving consideration to light scattering, and finger-vein information was used for recognition in various sizes and directions using an even-symmetric Gabor filter [20].

In [21], a method based on adaptive direct fuzzy contrast enhancement was optimized to improve the global contrast of images, and the Retinex theory was combined with optimal fuzzy conversion to improve the pattern information of local veins. Although this finger-vein recognition is able to obtain images easily and simply without touch through the use of near-infrared (NIR) light, its recognition performance is affected by different factors, such as illumination variations, finger positional variations, shading, misalignment, and quality changes that are caused by the pression of fingers over the sensor. According to this, studies have been conducted on recognition using the shape and geometry of fingers. Su et al. aligned fingers in the center after extracting the finger area from hand edges and extracting geometry descriptors. The geometrical features of each aligned finger were expanded using a wavelet transform. The image subtraction obtained was likewise used to conduct each genuine and imposter matching [22]. Asaari et al. used geometrical information while considering the finger width and fingertip angles for recognition. To extract robust features for motion and rotation, Fourier descriptors (FD) were used for feature extraction, and the orthogonality of the geometric information was improved through PCA. After this, matching was conducted using the Euclidian distance [23]. However, methods that consider finger geometry experience difficulties in extracting the features accurately when the fingers are too close together or too widely spread [22].

Recently, to make up for these shortcomings regarding the individual recognition of fingers, the multimodal recognition, which fuses the results of more than two finger-based recognition techniques, has come to the spotlight. Yang et al. proposed a fingerprint and finger-vein feature-level fusion-based multimodal recognition method that uses the supervised local-preserving canonical correlation analysis method (SLPCCAM) [24]. Peng et al. proposed a method for maximizing the score fusion through triangular norms (t-norms) by normalizing the scores of four types of features for finger-vein, finger shape, fingerprint, and finger knuckle-print score-level fusion [25]. In [26], before matching, recognition was attempted using methods that use feature-level fusion of homogeneous finger-vein, fingerprint, and finger knuckle-print features, and methods that use decision-level fusion using the results of applying support vector machine (SVM), or that use cosine distance and K-nearest neighbors (KNN) after conducting feature selection through kernel Fisher analysis (KFA), which applies PCA and LDA simultaneously. In [27], fractional firefly (FFF) optimization was used to find the optimal weight that expresses the best fused finger-vein and knuckle-print features. Moreover, classification was conducted by performing binary classification at each stage through layered k-support vector machine (K-SVM) combined with SVM and KNN. However, in [24], the experiment was conducted by obtaining fingerprint and finger-vein image separately using two devices, whereas in reality, using two devices would increase not only the size of the entire system but also its cost. Moreover, in [25,26,27], because the experiments were conducted by generating a multimodal database virtually from the existing finger-vein, finger shape, fingerprint, and finger knuckle-print databases, they do not apply when using images obtained from one finger of a real person. To solve these problems, in [28,29], a single device was used to obtain fingerprint, finger shape, and finger-vein images simultaneously. However, as recognition is conducted based on handcrafted features, there are limitations in terms of the performance improvement.

To overcome such limitations, this study proposes deep CNN-based finger-vein and finger shape multimodal biometrics that use finger-vein and finger shape information extracted simultaneously from finger images that were acquired with a single sensor-based single device using NIR light. Our research is novel in the following four ways compared to previous works.

- Convex polygons were generated using the algorithm that finds the coordinates of the outermost pixels of finger ROIs to calibrate the empty spaces of the images. Then, robust finger ROIs for misalignment were extracted after conducting in-plane rotation compensation based on the angle of tilting measured based on the boundaries of the upper, lower, left, and right pixels.
- Two-dimensional spectrogram images that express finger-thickness frequency-component changes depending on the horizontal position of fingers were obtained and used as CNN inputs for finger shape recognition.
- Matching distances calculated based on the features of finger-vein and finger shape that were obtained using ResNet models were score fused using various fusion methods, such as the weight sum, weighted product, and perceptron.
- The trained CNN models and algorithms developed in this study were open through [30] so that other researchers can use them in fair performance evaluations.

Table 1 shows the summarized comparisons on the proposed and previous studies on finger-based recognition.

The composition of this paper is as follows. Section 3 explains about the proposed multimodal finger-based recognition method. In Section 4 and Section 5, experimental results with analyses and the conclusion are provided, respectively.

## 3. Proposed Method

### 3.1. Overview of Proposed Method

**Figure 1:**
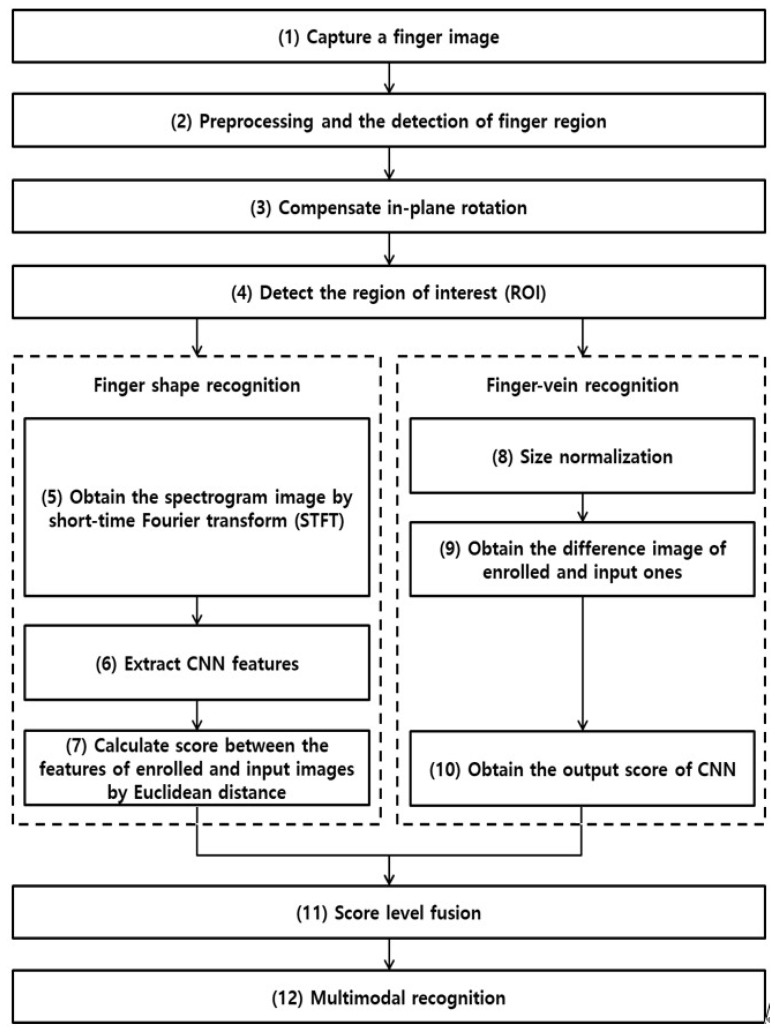
Flowchart of the proposed method.

The overall flowchart of the proposed method is shown in Figure 1. The captured finger images are binarized, and the binarized images are used to restore the collapsed areas through the convex hull algorithm [34] to extract accurate finger regions. Then, a 4 × 20 mask is used to detect the upper and lower boundaries to extract the finger regions more accurately than [18,32] (step 2 of Figure 1). After this, the in-plane rotation of the extracted finger region is calibrated (step 3) to obtain the final ROI image for CNN input (step 4). This ROI is used to find the spectrogram image (step 5), which is obtained by converting the vertical length changes with respect to the horizontal pixel position of the finger thickness. Then, the features of the finger shape are found using the CNN, which uses this spectrogram image as input (step 6). Next, the Euclidean distance between the input image features and the pre-enrolled image features is calculated and calibrated while using min-max scale normalization to obtain the score of finger shape (step 7). In addition, the ROI obtained in step 4 is normalized to the size of 224 × 224 (step 8), and the pixel-difference images from the pre-enrolled image are found to calculate matching scores through the CNN, which uses such pixel-difference images as input. Then, these scores are calibrated using mix-max scale normalization to obtain the score of the finger-vein (steps 9 and 10). The two scores obtained through steps 7 and 10 are score-level fused (step 11) to finally conduct multimodal recognition of the finger shape and finger-vein (step 12).

### 3.2. Preprocessing and Detection of Finger Region

**Figure 2:**
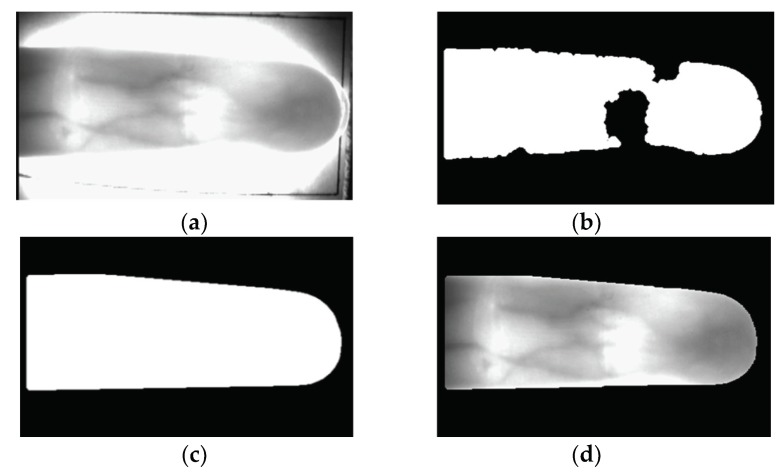
Examples of input image and detected finger region: (**a**) original input image; (**b**) incorrect binarized image; (**c**) corrected binarized image by component labeling and convex hull processing; and, (**d**) detected finger region.

The Sobel operator and component labeling are applied to the captured finger images for noise removal. Then, binarized images, such as that shown in Figure 2b, are obtained through image thresholding. However, as shown in Figure 2b, incorrect binarized images are obtained often due to non-uniform illumination inside the finger area. To solve this, the convex hulls are found and calibrated [34]. Convex hulls are minimum-size convex polygons containing all areas. To set the part to be calculated, the height and width of the x coordinate, y coordinate, and finger-vein area of the top-left of the binarized images are calculated to set the bounding box that contains all of the finger-vein areas. The outermost pixel coordinate of the bottom-right area inside the bounding box containing binarized areas is determined as a starting point for convex hull construction. The outermost coordinate points of pixels with the furthest distance counter-clockwise from the outermost pixel coordinate’s starting point are considered boundary areas and are connected together. Here, the angle from the outermost pixel coordinate to the next outermost pixel coordinate is clockwise, but nearby coordinate points are ignored. This is repeated until the process returns to the starting point; all the pixels inside the area are calculated and all of the pixels inside convex polygons are filled to calibrate the eroded area inside of fingers [34]. Through this, as shown in Figure 2c,d, accurate finger regions without eroded areas inside are obtained.

### 3.3. In-Plane Rotation Compensation

**Figure 3:**
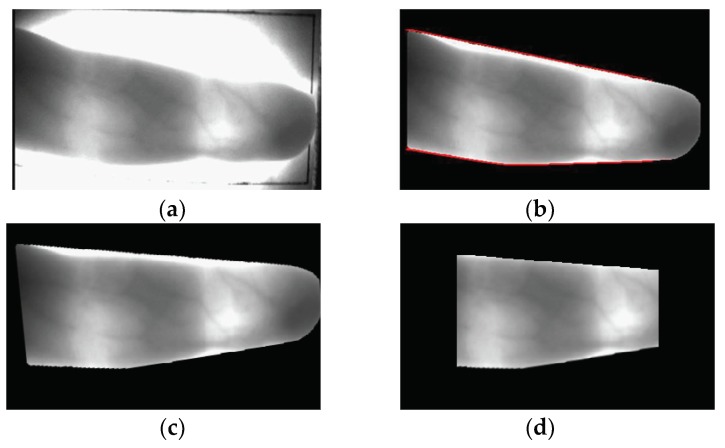
Examples of in-plane rotation compensation: (**a**) original input image; (**b**) extracted finger region by step 2 of Figure 1; (**c**) image by in-plane rotation compensation; and, (**d**) detected finger region-of-interest (ROI).

Misalignment between enrolled and recognized images hinders matching and causes low recognition performance. In this research, to solve these problems, in-plane rotation compensation was conducted on the input finger images. As shown in Figure 2d, for an accurate rotation angle measurement in the obtained finger images, a 4 × 20 mask was used to detect the upper and lower boundaries of finger-veins, as shown in the red-colored lines of Figure 3b [18,32]. After this, the two-dimensional moment of the finger region between these two boundaries is calculated and used for in-plane rotation compensation [35]. Because there are many cases where the left and right ends of the compensated finger regions contain areas unsuitable for recognition due to uneven illumination that is caused by nails and finger thickness, the final finger ROIs are detected, as shown in Figure 3d, after removing a defined portion of the left and right ends. These ROIs are used for finger shape and finger-vein recognition.

### 3.4. 2-Dimensional Spectrogram Image for Finger Shape Recognition

**Figure 4:**
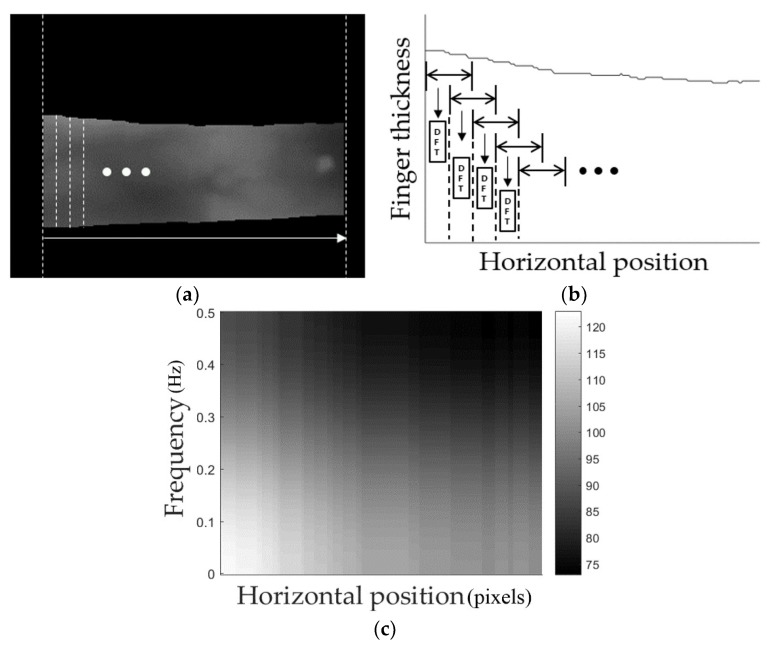
Examples of spectrogram image of finger shape: (**a**) finger ROI image; (**b**) applying short-time Fourier transform (STFT) on one-dimension graph of finger thickness; and, (**c**) two-dimensional spectrogram image.

In this research, finger shape recognition was conducted after finding two-dimensional spectrogram images (Figure 4) based on the finger thickness in the horizontal position. In various signal-processing and -recognition fields containing speech recognition, spectrograms have been widely used to extract meaningful features [36,37,38,39,40,41,42]. Spectrograms are general methods to indicate the corresponding signals in time–frequency areas. In this study, as shown in Figure 4a,b, the finger region-of-interest (ROI) thickness in the horizontal position is measured and expressed with a one-dimensional graph to obtain the finger shape information. The frequency components were extracted using short-time Fourier transform (STFT) (Equation (1)) at each location through sliding by overlapping by 1 pixel in a fixed-size window on this graph [37,40].

(1) $$\mathrm{STFT}\left(n,w\right)=\sum_{m=0}^{R-1}\left(x\left[m\right]W\left[n-m\right]\right)e^{-i2\pi wm}.$$

Here, *n* is the horizontal position of the finger ROI, *w* is the frequency, *x*[*m*] is the signal to be analyzed, and *W*[m] is a window. Through this, the two-dimensional spectrogram, which is the magnitude of the frequency of each horizontal position, was found, as shown in Figure 4c, and used as input images for CNN. This two-dimensional spectrogram image expresses the difference in the frequency amplitude of the horizontal position axis as a color-density difference. That is to say, as shown in Figure 4c, greater finger-thickness changes inside the corresponding window are indicated by brighter high-frequency areas, and smaller changes are indicated by brighter low-frequency areas.

Generally, because CNN structures use one image as input, to conduct recognition using enrolled and input images as in this research, each feature vector of both images in the layer before the CNN’s fully connected layer was extracted to calculate the distance between the feature vectors of these two images and determine which one is genuine and which is an imposter [43]. In this study as well, two feature vectors of the CNN, which use the two two-dimensional spectrogram images that were obtained in the enrolled and input images (such as in Figure 4c), were found and used for genuine- and imposter-matching based on the Euclidean distance between them.

### 3.5. Difference Image Finger-Vein Recognition

**Figure 5:**
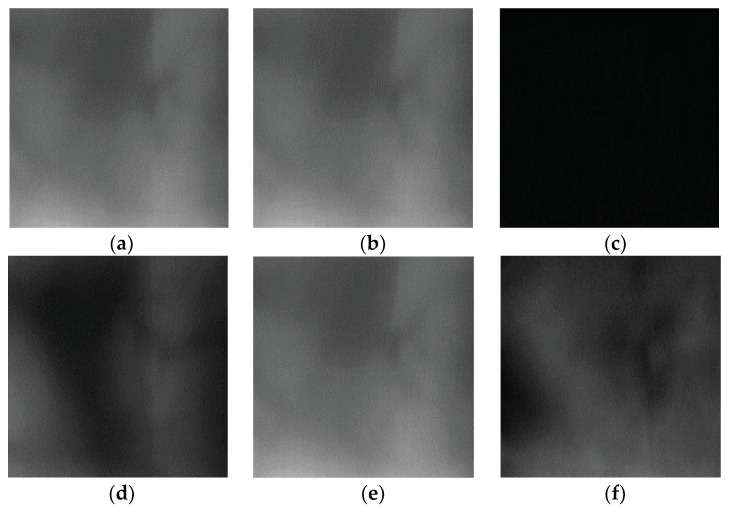
Examples of input and enrolled images with their corresponding difference images: (**a**) input image and (**b**) enrolled image in same class; (**c**) difference image of (**a**) and (**b**); (**d**) input image and (**e**) enrolled image in different class; and, (**f**) difference image of (**d**) and (**e**).

In previous CNN-based finger-vein recognition studies, instead of a method that determines the genuine or imposter by extracting the feature vectors of two images in the CNN’s fully connected layer or the layer before that, then finding the distance between these two feature vectors, a method using one difference image found in both the enrolled and input finger-vein images as the CNN input image was found to provide a higher recognition accuracy [32]. Based on these results, in this study, one different image (Figure 5c,f) obtained from the finger ROI image of the enrolled and input images found, as shown in Figure 3d, was used as the CNN input. As shown in Figure 5, the difference image of two images of the same class shows a dark pixel value due to the difference between the pixels of both images being small (Figure 5c, and the difference image of two images of different class shows a relatively bright pixel value due to the difference between the pixels of both images being large (Figure 5f). Genuine and impostor matches are finally determined based on the CNN output result.

### 3.6. CNN-Based Finger-Vein and Finger Shape Recognition

**Figure 6:**
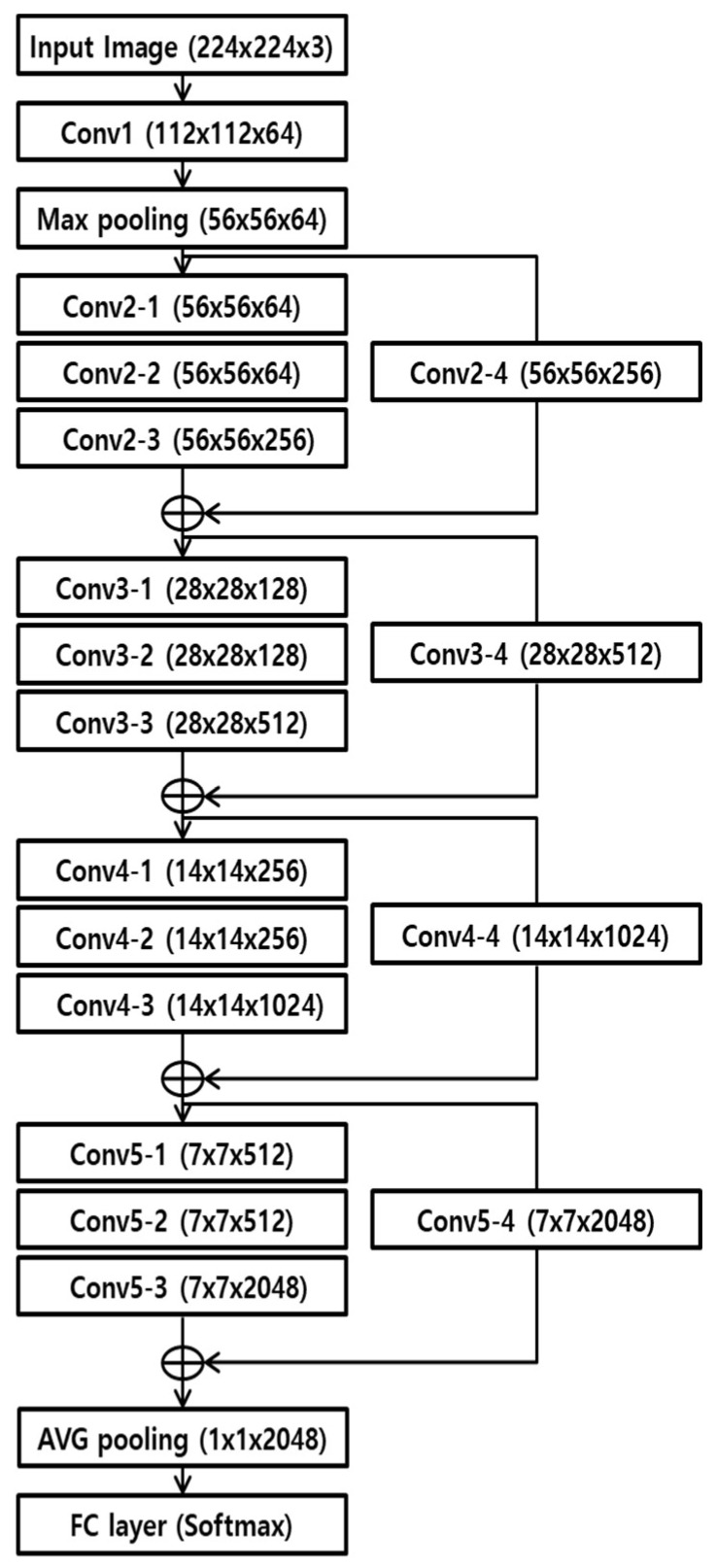
Convolutional neural network (CNN) structure used in our research.

In this study, the same structure of ResNet-50 and ResNet-101 [44] was used, excluding the output node. The experiment was conducted by fine-tuning the training data used in this study. In other words, for finger-vein recognition, both genuine and imposter matches were set as the output class. Additionally, for finger-vein recognition, the number of classes contained in the training data was set as the number of CNN output nodes. Figure 6 and Table 2 show the structure of the RestNet CNN model that was used in this study. According to the proposed structure, the input images were resized to 224 × 224 pixels. The feature map size output based on the input image is calculated, as follows: Feature map width (or height) = (Input width (or height) − kernel width (or height) + number of padding pixels × 2)/number of strides + 1 [45]. For example, in Table 2, the feature map width (or height) output in the first convolutional layer (Conv1) is 112 (= (224 − 7 + 3 × 2)/2 + 1). For structure optimization, batch normalization and rectified linear units (ReLU) are passed through each convolutional layer. During batch normalization, the mean and covariance of the features are found and normalized in uncorrelated mini-batch units [46]. The following is the equation of normalization of the mean and covariance of mini-batches.

(2) $${\hat{x}}_{i}=\frac{x_{i}-\mu_{m}}{\sigma_{m}}.$$

Here, $x_{i}$ is the mini-batch data consisting of n units, $\mu_{m}$ is the mean of the corresponding mini-batch, $\sigma_{m}$ is the square-root value of covariance of the corresponding normalized mini-batch, and ${\hat{x}}_{i}$ is the corresponding normalized mini-batch. Through the internal covariate shift of Equation (3), this can prevent vanishing gradient problems that fall in the local minimum during learning due to weight changes in the hyperparameters of all the data with the same learning rate [46].

(3) $$\mathrm{BN}\left(x_{i}\right)=\gamma {\hat{x}}_{i}+\beta .$$

Here, BN means batch normalization, γ is the scale and β is the shift parameter. After this, as shown in Equation (4), ReLU functions, which only process negative values as 0, were used as activation functions to improve the training convergence speed more than when using sigmoid functions [47].

*y* = max (0, *x*), (4)

where *x* and *y* are the input and output of a ReLU function, respectively. Because the output range of *y* can be reduced to 0 or a positive value, the ReLU function can be partially or sparsely activated, and can thus facilitate the training of the CNN model. The mathematical equation for training becomes simpler and can prevent the vanishing gradient problem [47]. Moreover, after the first convolutional layer, it acts as subsampling, using the greatest value within a 3 × 3 window area through 3 × 3 max pooling.

In ResNet CNN, a bottleneck structure is used [44]. As shown in Table 2, first, the feature map dimension was downscaled in a 1 × 1-size convolution layer, and features were extracted in a 3 × 3-size convolution layer. To connect the previous feature map with the feature map that passed through a shortcut path that is transmitted as-is to the following layer, a 1 × 1-size convolution layer was used again in the following convolution layer to expand it to the same size of the feature map dimension that passed through the shortcut path. Through this bottleneck structure, the number of learning parameters decreases more than in a single 3 × 3-size convolution process, thus reducing the amount of computation that is required [44]. By transmitting the previous feature map as-is to the following layer using a shortcut path, which is an important characteristic of ResNet structures, the loss of important residual information of the feature map, which is lost during convolution processing, can be somewhat reduced. This feature map, which has passed through convolutional layers, finally goes through an average pooling, which processes the average value within a window and recognizes the label class through a fully connected layer. Due to this, degradation problems occurring when the layers become deep are solved and the learning is optimized.

The softmax function [48] can be applied to the fully convolutional layer output, as shown in Equation (5).

(5) $$\sigma {\left(s\right)}_{j}=\frac{e^{sj}}{\sum_{n=1}^{K}e^{sn}},$$

when the array of output neurons is set to *s*, the probability of the neurons belonging to the *j*th class is obtained by dividing the value of the *j*th element by the sum of the values of all elements.

### 3.7. Finger Recognition Based on Score-Level Fusion

As shown in step (11) of Figure 1, the two matching scores obtained through the two CNNs for finger shape and finger-vein recognition are combined through score-level fusion to obtain the final matching score. In other words, as explained before, in finger-vein recognition, the scores of genuine and imposter matches in the fully connected layer of Table 2 are calculated and used, and in finger shape recognition, 2048 features in the average pooling layer of enrolled and input images are extracted, and the Euclidean distance between these 2048 features is used as the matching distance.

As the scales of these two scores are different, it is necessary to convert them to the same numerical range. Min-max normalization is conducted while using the maximum and minimum values of the scores that were extracted based on training data from each recognition method to convert the score range into a number between 0 and 1. The two normalized matching scores were used to compare the recognition performance applying score-level fusion based on the weighted sum, weighted product, Bayesian rule, and perceptron rule [49], as shown in Equations (6)–(9).

(6) $$S_{ws}=w\times S_{1}+\left(1-w\right)\times S_{2},$$

(7) $$S_{wp}=S_{1}{}^{w}\times S_{2}{}^{1-w},$$

(8) $$S_{B}=\frac{S_{1}S_{2}}{\left(1-S_{1}\right)\left(1-S_{2}\right)+\left(S_{1}S_{2}\right)},$$

(9) $$S_{P}=\frac{1}{1+e^{-\left(w0+w1S_{1}+w2S_{2}\right)}}.$$

Here, $S_{1}$ and $S_{2}$ are the scores of finger-vein and finger shape recognition, respectively. w, w0, w1, and w2 represent the weighted values. The sum of w0, w1, and w2 is always 1. Based on the final score obtained through score-level fusion, if the score is higher than the threshold, it is considered an imposter, and if it is lower, it is considered to be a genuine match.

## 4. Experimental Results

### 4.1. Experimental Data

**Figure 7:**
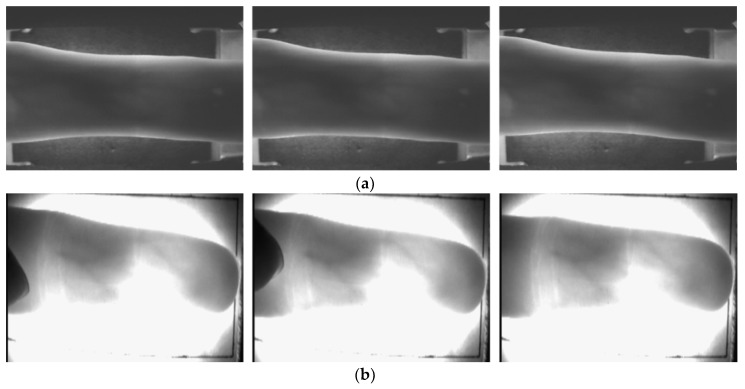
Examples of input images of different trials from the same finger of one individual from each database: (**a**) SDU-DB; (**b**) PolyU-DB.

In this study, to verify the robustness of the proposed method under severe finger database noise or when a part of the finger-vein area is damaged, two open databases were used. The first database was the Shandong University homologous multi-modal traits (SDUMLA-HMT) finger-vein database. This database is composed of six images from the index, middle, and ring fingers of both hands of 106 people for a total of 3816 images (106 people × 2 hands × 3 fingers × 6 images) [50]. The second database is the Hong Kong Polytechnic University Finger Image Database (version 1), which is composed of two sessions [35]. The first session is composed of six images from the index and middle fingers of one hand of 156 people for a total of 1872 images (156 people × 2 fingers × 6 images). The second session is composed of six images from the index and middle fingers of one hand of 105 people from the 152 people of the first session for a total of 1260 images (105 people × 2 fingers × 6 images). In this study, the experiment was conducted using the database of the first session. Figure 7 shows an example of the database used in this study. From now on, the SDUMLA-HMT database shall be referred to as SDU-DB, and the Hong Kong Polytechnic University Finger Image Database (version 1) shall be referred to as PolyU-DB.

SDU-DB is composed of 636 (106 × 2 × 3) classes and PolyU-DB is composed of 312 (156 × 2) classes. The data of the different classes were tested using two-fold cross-validation to be included in training and testing data. In other words, in the first validation, the images of 318 classes from SDU-DB were used in training, and the remaining 318 were used in test. Similarly, the images of 156 classes of the PolyU-DB were used in training, and the remaining 156 classes were used in test. Then, in the second validation, the data used in training and testing were interchanged to conduct training and testing one more time. The mean accuracy that was obtained through this double experiment was used as the final recognition accuracy.

### 4.2. Data Augmentation

It is difficult to learn the many parameters and weights within the deep CNN structure that is used in this study with only the training data explained in Section 4.3, and overfitting problems occur. To solve these problems, the number of classes was maintained and the pixel position of the training images of each class was converted using translation and cropping to conduct data augmentation, which is a method that is used to increase the amount of training data. This data-augmentation method based on image translation and cropping has been widely used in previous studies [51].

Finger shape images were translated and cropped by 1–5 pixels at a time in input images in four directions (up, down, left, and right), and then resized to obtain 121 images. As described before, in SDU-DB, 318 classes were used in training and 318 were used in test. Because six images exist in each class in the training data of 318 classes, they were augmented 121 times through data augmentation for a total of 230,868 (318 × 6 × 121) images. Similarly, in PolyU-DB, six images exist in each of the 156 classes of the training data, which were augmented 121 times as well for a total of 113,256 (156 × 6 × 121) images.

Finger-vein images are divided into two different cases of the generation of training images for genuine and imposter matching. In SDU-DB, when generating training images for genuine matching, first, the six images of each class are translated and cropped by 1–4 pixels at a time randomly in the horizontal and vertical directions and then resized, augmenting them by 13 for a total of 78 images. Then, 77 difference images between the one image to be used for enrollment and the remaining 77 images are obtained. This process is repeated for all of SDU-DB’s training classes (318 classes) to finally obtain a total of 24,486 ((6 × 13 − 1) × 318) training images. When generating training images for imposter matching, because the number of difference images between the images of the different classes is significantly more than the number of training images that is used for genuine matching, a total of 24,486 images are selected through random selection from all of the difference images to balance the number of training images for genuine matching and imposter matching. Through this, the CNN training is prevented from being biased towards any side. In PolyU-DB as well, the same method is used to obtain 12,012 ((6 × 13 − 1) × 156) training images for genuine matching along with the same amount for imposter matching. The description of the databases and augmented images is shown in Table 3. This data-augmentation process was conducted only for training data. For the testing data, non-augmented original images were used.

In this study, training and testing were conducted in a desktop computer environment (Intel^®^ Core™ i7-6700 CPU @ 3.4 GHz (4 cores) (Intel, Santa Clara, CA, USA) with 32 GB of RAM, and NVIDIA GeForce GTX Titan X (3072 CUDA cores) (Nvidia, Santa Clara, CA, USA) with graphics memory of 12 GB [52]). The algorithm was implemented using Caffe Framework [53] and Microsoft Visual Studio 2013 [54].

### 4.3. Training of CNN

**Figure 8:**
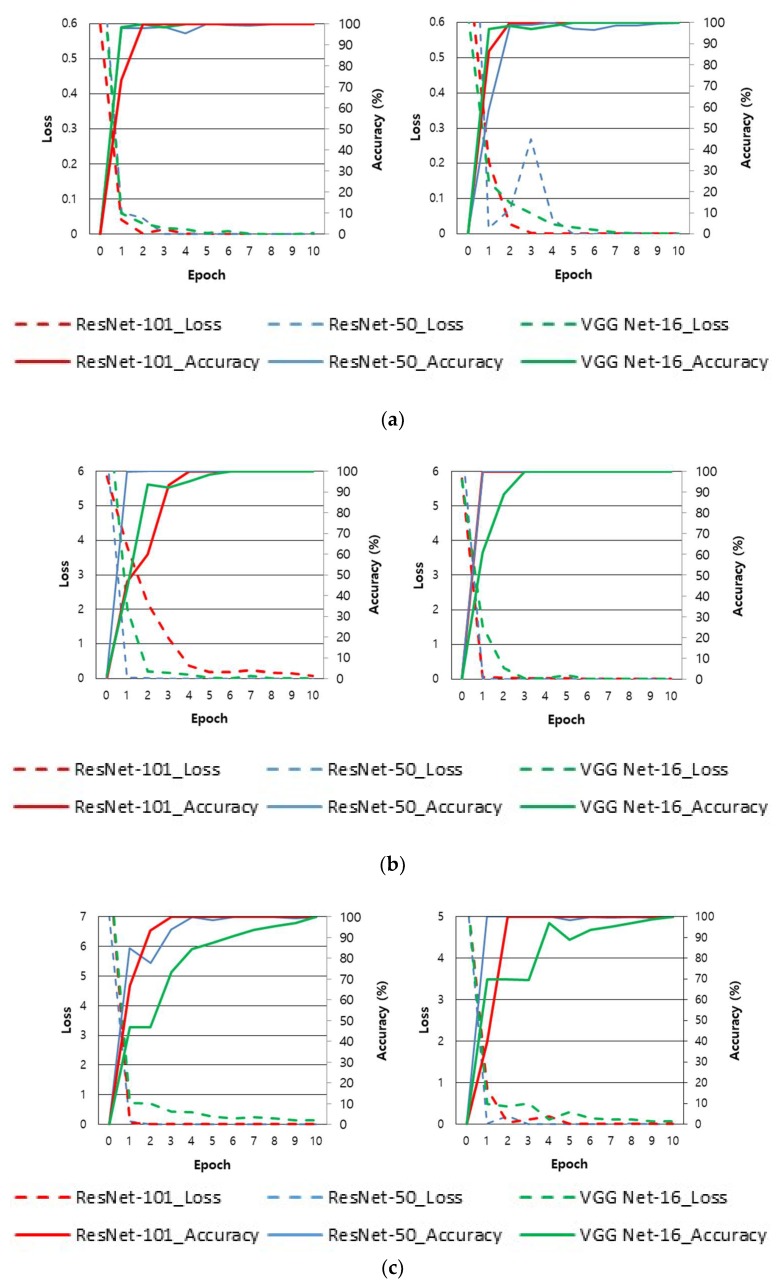

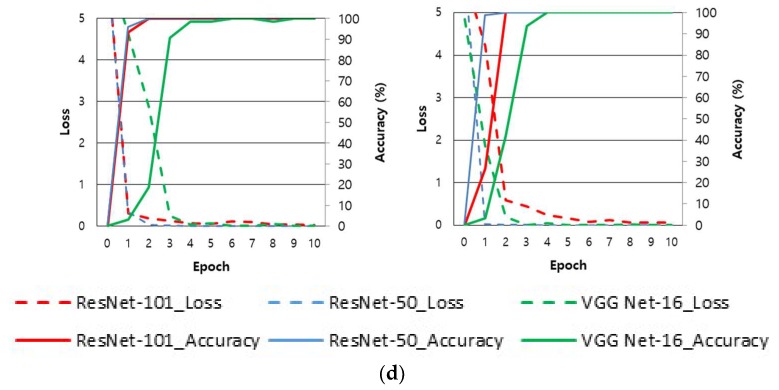
Examples of loss and accuracy curves with training data of two-fold cross validation according to databases. Using (**a**) finger-vein images in SDU-DB database, (**b**) finger shape images in SDU-DB database, (**c**) finger-vein images in PolyU-DB database, and (**d**) finger shape images in PolyU-DB database. In (**a**)–(**d**), the left and right figures show the results of the first and second cross validation, respectively.

The data-augmented input images were used to conduct learning using the proposed CNN structure. For optimized CNN learning, the stochastic gradient descent (SGD) method was used [55]. This SGD method rapidly converges the training accuracy and loss while reducing the learning rate with the value that was calculated by multiplying gamma values step by step so that the training does not diverge at each epoch by mini-batch units. In this experiment, it was verified that convergence occurs rapidly when using a learning rate of 0.001, a momentum of 0.9, a gamma of 0.1, a mini-batch of 20 for ResNet-50 (15 for ResNet-101), and a maximum epoch of 10. After five epochs, the learning rate was reduced. The result of dividing the entire training data by the mini-batch size is called the number of iterations. One epoch is when as much training has been performed as the total number of iterations. Therefore, the total number of trainings is equivalent to the number of iterations × epoch. Table 4 shows an explanation of the initial parameters used during CNN training, and Figure 8 shows the training accuracy and loss of the VGG Net-16 [56] used for ResNet CNN and performance comparison in this study. The x axis in Figure 8 shows the number of epochs, and the y axis shows the training loss and accuracy of each epoch. As shown in Figure 8, through training, the accuracy converges near 100 and the loss becomes nearer to 0.

### 4.4. Testing of Proposed CNN-Based Recognition

#### 4.4.1. Comparison of the Accuracy of Finger-Vein Recognition

The finger-vein recognition accuracy was measured through the first experiment. As explained in Section 4.1, all of the experiments were conducted with two-fold cross-validation, and the average value of both testing errors were shown. The false acceptance rate (FAR) and false rejection rate (FRR) are the methods that are used for recognition error rate measurement. FAR shows the error rate of incorrectly judging different classes as genuine matches, and FRR shows the error rate of incorrectly judging equal classes as imposter matches. In FAR and FRR, when both classes have trade-off characteristics, the error at the moment when FAR and FRR become the same is called the equal error rate (EER).

As shown in Table 5, the EER of the method that is proposed in this study is 3.3653% for SDU-DB and 1.0779% for PolyU-DB. Moreover, the accuracy of the method proposed in this study and the accuracy of the methods proposed in previous studies were compared. As for the previous methods, the non-training-based Gabor filtering, maximum curvature [13], repeated line tracking [15], wide line detector [16], and Gabor filtering + LBP [18] were compared with the VGG Net-16-based finger-vein recognition method [32], which is training-based. As shown in Table 5, it was confirmed that the accuracy of the method proposed in this study is higher than that of previous methods.

**Figure 9:**
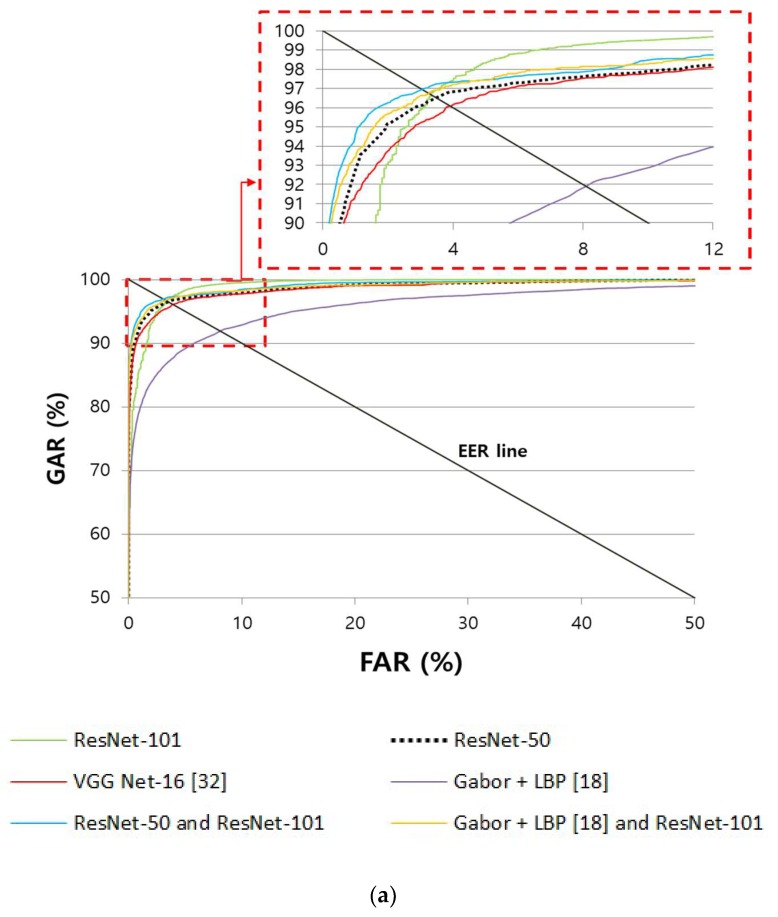

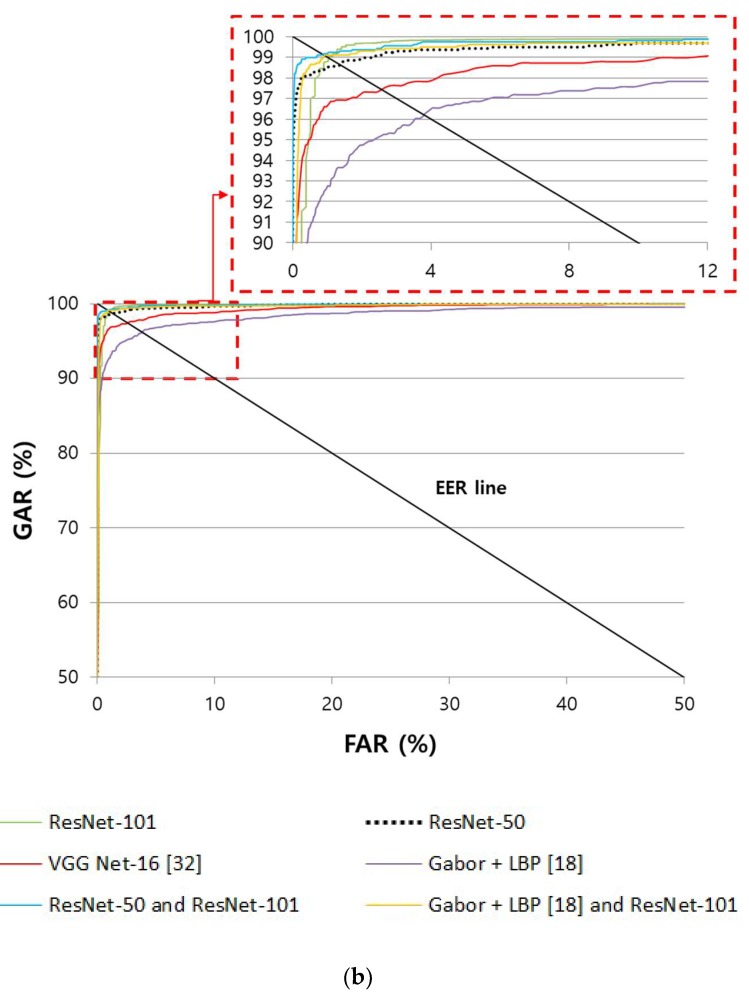
Comparison of receiver operating characteristic (ROC) curves of finger-vein recognition with (**a**) SDU-DB and (**b**) PolyU-DB.

Moreover, Figure 9 shows the receiver operating characteristic (ROC) curves. Here, the genuine acceptance rate (GAR) is calculated as 100−FRR (%). As described before, this was expressed as the mean curve of the two ROC curves obtained through two-fold cross-validation. As shown in Figure 9, it was confirmed that the accuracy of the finger-vein recognition method proposed in this study is higher than that of existing methods.

**Figure 10:**
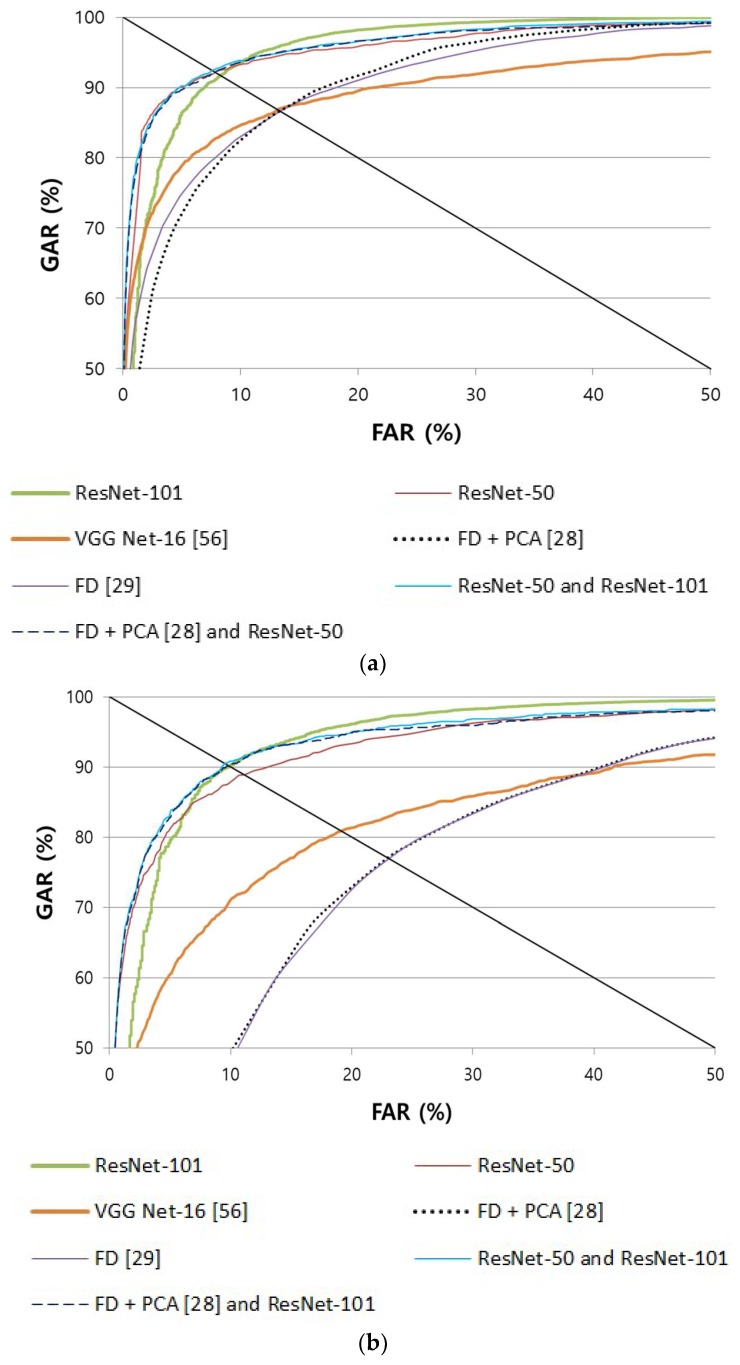
Comparison of ROC curves of finger shape recognition with (**a**) SDU-DB and (**b**) PolyU-DB.

In addition, we included the additional experimental results by combining the different CNNs in Table 5 and Table 6 and Figure 9 and Figure 10. Because the ResNet-50 and ResNet-101 show the better accuracies than other model, as shown in Table 5 and Table 6, the results by ResNet-50 and ResNet-101 models are combined by score-level fusion of weighted sum, weighted product, Bayesian, and perceptron rules of Equations (6)–(9) after score normalization according to comment. Experimental results showed that the weighted product rule shows the higher accuracy than other rules in both cases of finger-vein and finger shape recognition. Based on them, we included the accuracies by the weighted product rule in Table 5 and Table 6 and Figure 9 and Figure 10. The accuracies by this score-level fusion are a little higher than those only by ResNet-50 and ResNet-101, as shown in Table 5 and Table 6 and Figure 9 and Figure 10. However, the accuracies by the score-level fusion are lower than those by our method based on the combination of finger-vein and finger shape recognition using either ResNet-50 or ResNet-101. In addition, the processing time increases by executing both ResNet-50 and ResNet-101 for the score-level fusion compared to that by our method.

As the next test, we included the additional experimental results by combining trained and non-trained based methods in Table 5 and Table 6 and Figure 9 and Figure 10. In the case of finger-vein recognition, the results by Gabor + LBP [18] and ResNet-101 are combined by score-level fusion of weighted sum, weighted product, Bayesian, and perceptron rules of Equations (6)–(9) after score normalization according to comment. In case of finger shape recognition, the results by FD + PCA [28] and ResNet-50 for SDU-DB (ResNet-101 for PolyU-DB) are combined by score-level fusion of weighted sum, weighted product, Bayesian, and perceptron rules of Equations (6)–(9) after score normalization. Experimental results showed that the weighted product rule shows the higher accuracy than other rules in both cases of finger-vein and finger shape recognition. Based on them, we included the accuracies by the weighted product rule in Table 5 and Table 6 and Figure 9 and Figure 10. The accuracies by this score-level fusion are a little higher than those only by ResNet-50 and ResNet-101, as shown in Table 5 and Table 6 and Figure 9 and Figure 10. However, the accuracies by the score-level fusion are lower than those by our method based on the combination of finger-vein and finger shape recognition using either ResNet-50 or ResNet-101, respectively. In addition, the processing time increases by executing both ResNet model and non-trained method for the score-level fusion when compared to that by our method.

#### 4.4.2. Comparison of the Accuracy of Finger Shape Recognition

In the second experiment, the accuracy of finger shape recognition was measured. For finger shape recognition, spectrogram images were generated in both input and enrolled images, as explained in Section 3.4, to extract 2048 features from the average (AVG) pool layer of Table 2. Then, the Euclidian distance between these features was calculated. The thereby calculated distance value was used to measure the recognition performance through genuine and imposter matching. The method that was proposed in this study was compared against the non-training-based FD method used in a previous study [29], which expresses the thickness data of the finger-vein horizontal length as a frequency band, and against the existing method [28], which combines FD and PCA. Moreover, to compare the performance of different CNN models, the accuracy obtained when using VGG Net-16 [16] was also compared based on the same spectrogram images that were used in this research. As shown in Table 6 and Figure 10, it can be known that the recognition performance of the method used in this study is higher than the accuracy of the existing method and the accuracy obtained when using VGG Net-16.

**Figure 11:**
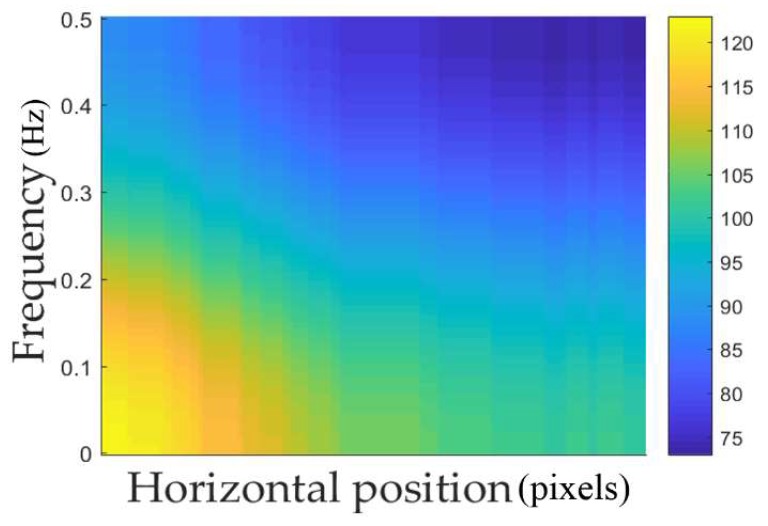
Example of 3 channel color spectrogram image of Figure 4a,b.

In the next experiment, we compared the accuracies of finger shape recognition based on one channel gray spectrogram image and three channels color spectrogram image of Figure 11 because the three channels color spectrogram image has been widely used in previous researches [38,39,40,42]. As shown in Table 7, the method using one channel gray spectrogram image outperforms that using three channel color spectrogram image on both SDU-DB and PolyU-DB. The reason why the method using three channel color spectrogram image shows lower accuracy is as follows. The three channel color spectrogram image presents the frequency information by pseudo-colors, as shown in Figure 11, and the level of discontinuity in this presentation is larger than that in one channel gray spectrogram image using continuous gray level of Figure 4c, although the human visibility in three channel color spectrogram image is better. This discontinuity causes the generation of incorrect filters of CNN, which can reduce the recognition accuracy.

#### 4.4.3. Comparison of the Accuracy of Multimodal Recognition of Finger-Vein and Finger Shape According to Various Score-Level Fusion Methods

**Figure 12:**
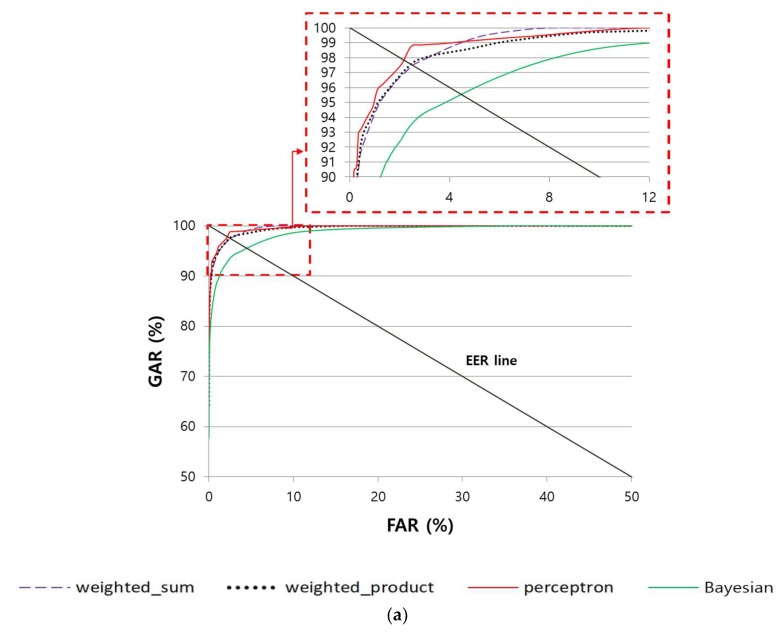

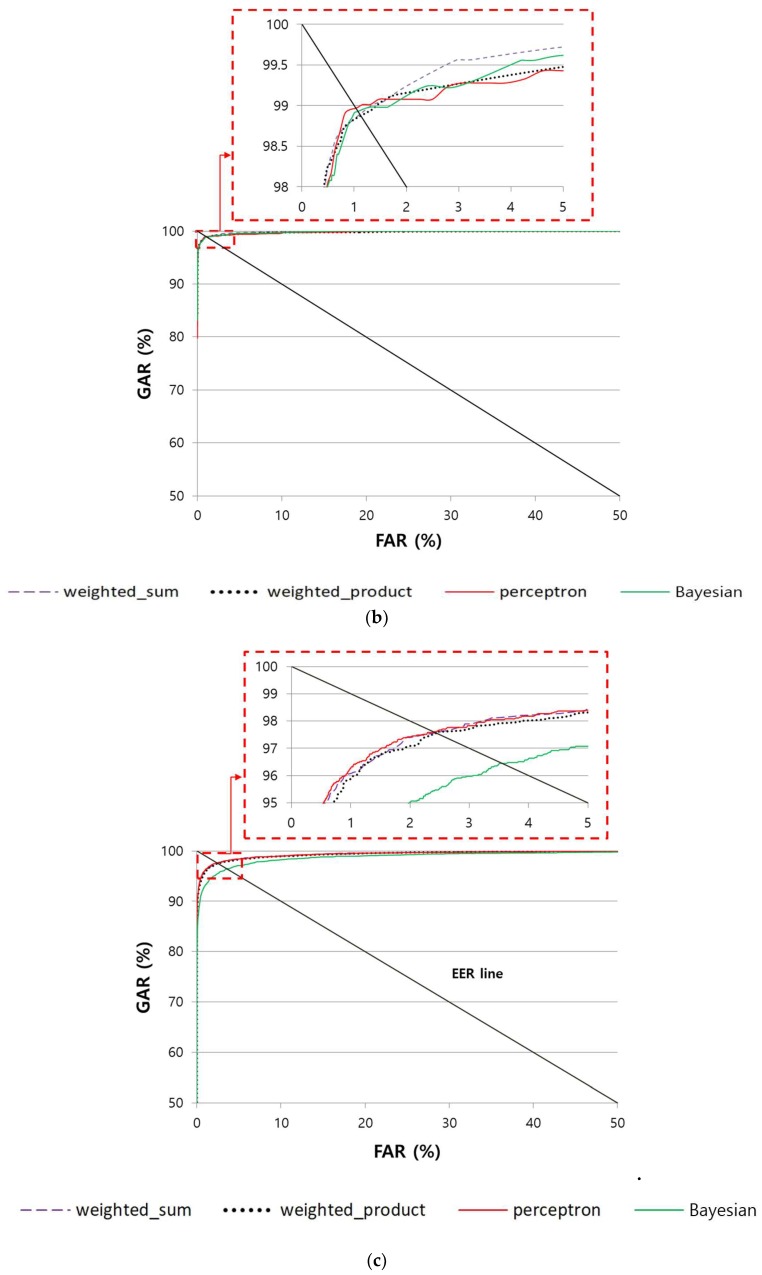

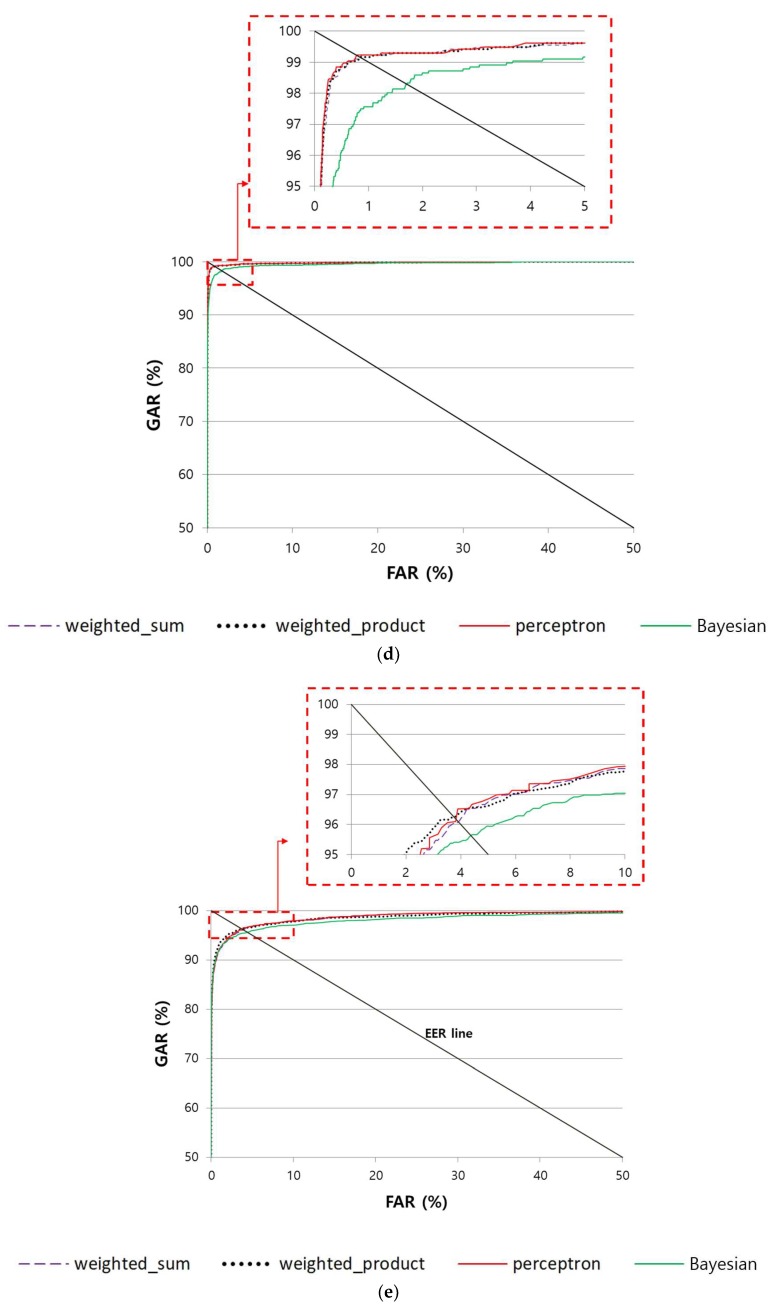

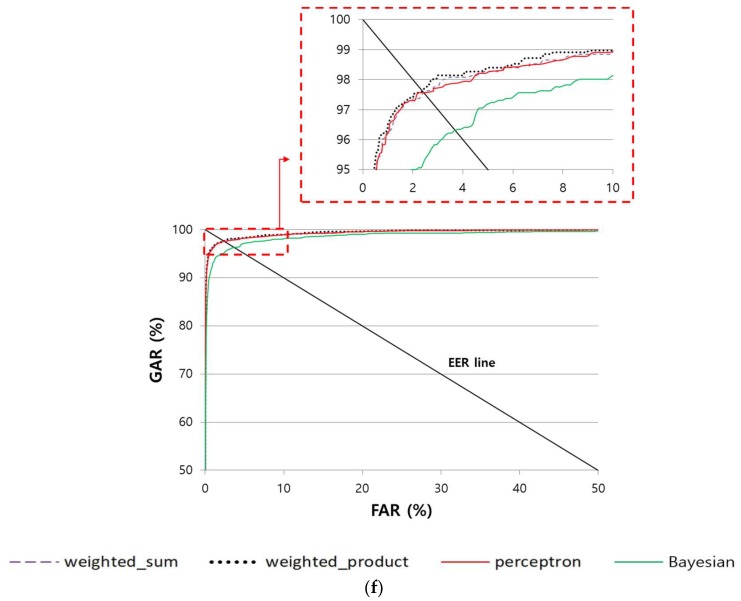
Comparison of ROC curves of multimodal recognition according to various score-level fusions in the case using (**a**) ResNet-50 with SDU-DB, (**b**) ResNet-50 with PolyU-DB, (**c**) ResNet-101 with SDU-DB, (**d**) ResNet-101 with PolyU-DB, (**e**) VGG Net-16 with SDU-DB, and (**f**) VGG Net-16 with PolyU-DB.

In the following experiment, the recognition performance of the various score-level fusion methods explained in Section 3.7 was compared against the finger-vein and finger shape recognition methods that were proposed in this study. Moreover, to compare the performance of various CNN models, the accuracy obtained when VGG Net-16 was used [56], not the ResNet method used in this study, was also compared. As shown in Table 8 and Figure 12, the perceptron rule-based method displayed the best performance, and SDU-DB and PolyU-DB displayed an EER of 2.3445% and 0.7859%, respectively.

**Figure 13:**
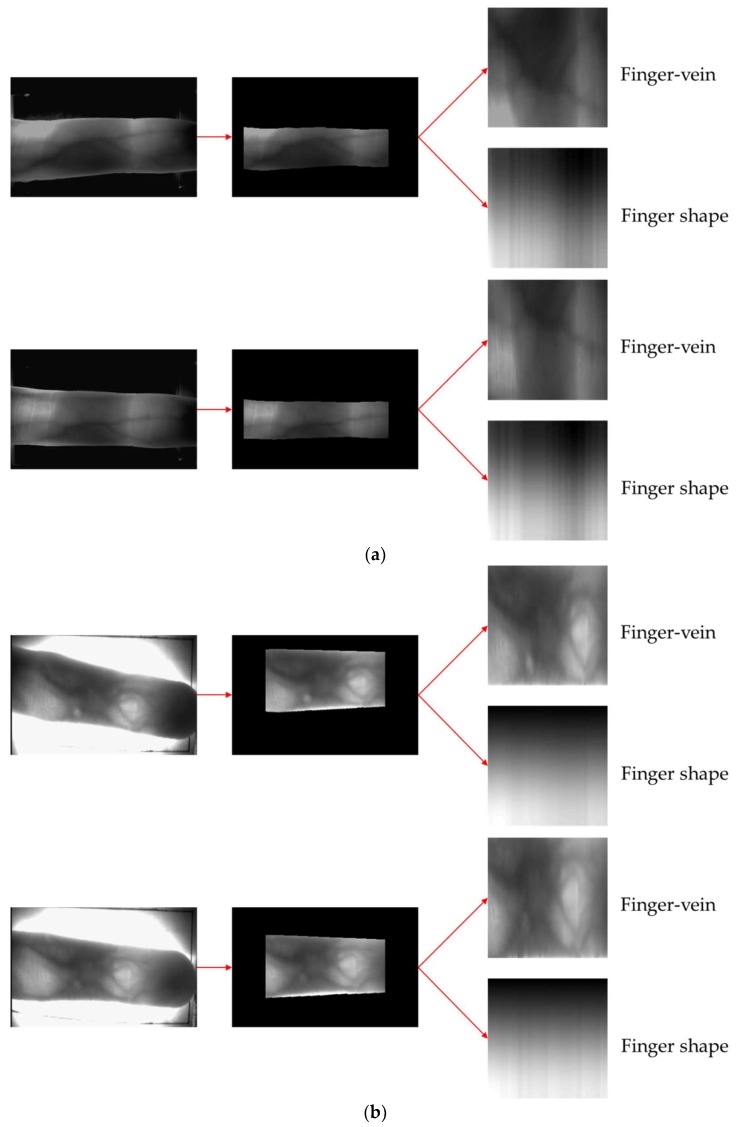

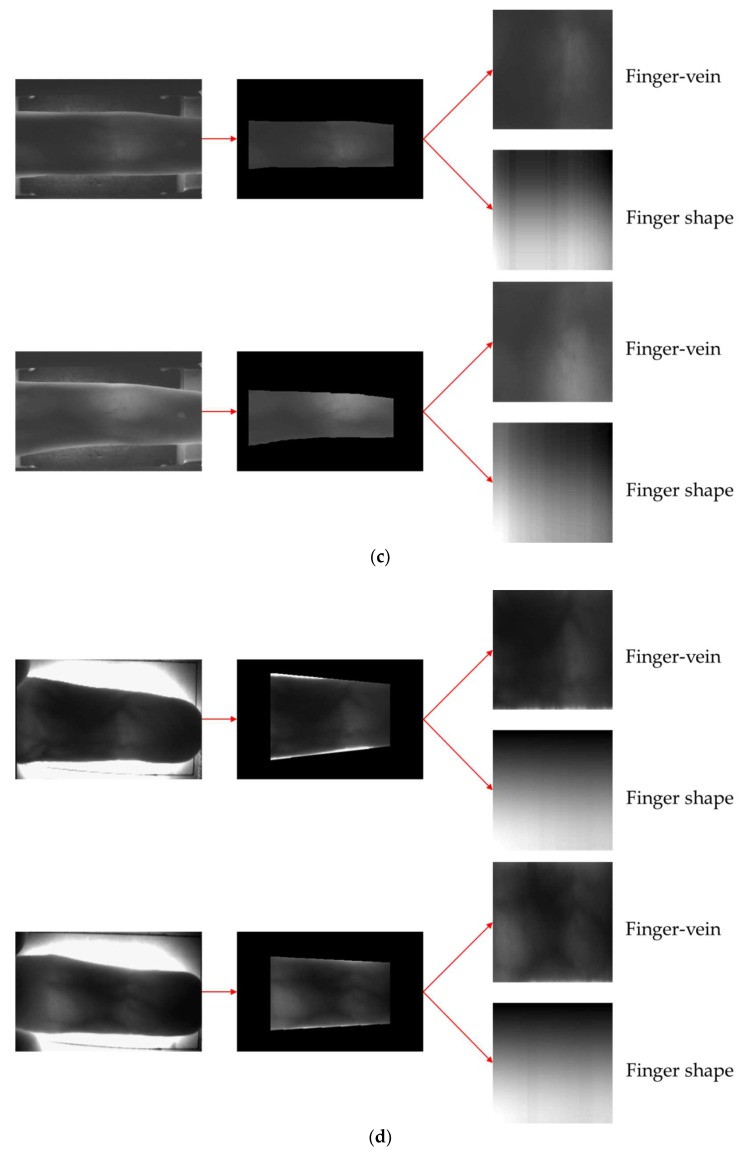
Examples of the correct recognition cases. (**a**,**b**) the correct acceptance cases with SDU-DB and PolyU-DB, respectively. (**c**,**d**) the correct rejection cases with SDU-DB and PolyU-DB, respectively. In (**a**–**d**), upper and lower images show the enrolled and input images, respectively. In (**a**–**d**), left and middle images present the input and corresponding finger ROI. The right-upper images show that for obtaining the difference image for finger-vein recognition, whereas the right-lower images present the spectrogram images for finger shape recognition**.**

**Figure 14:**
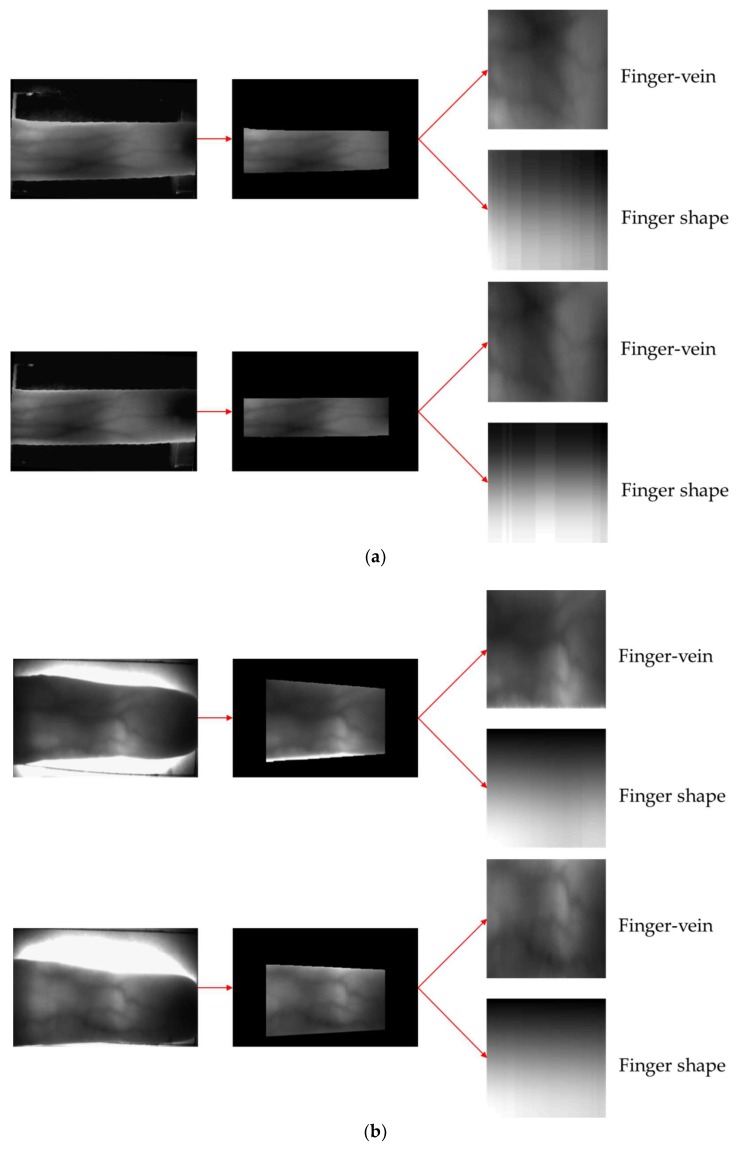

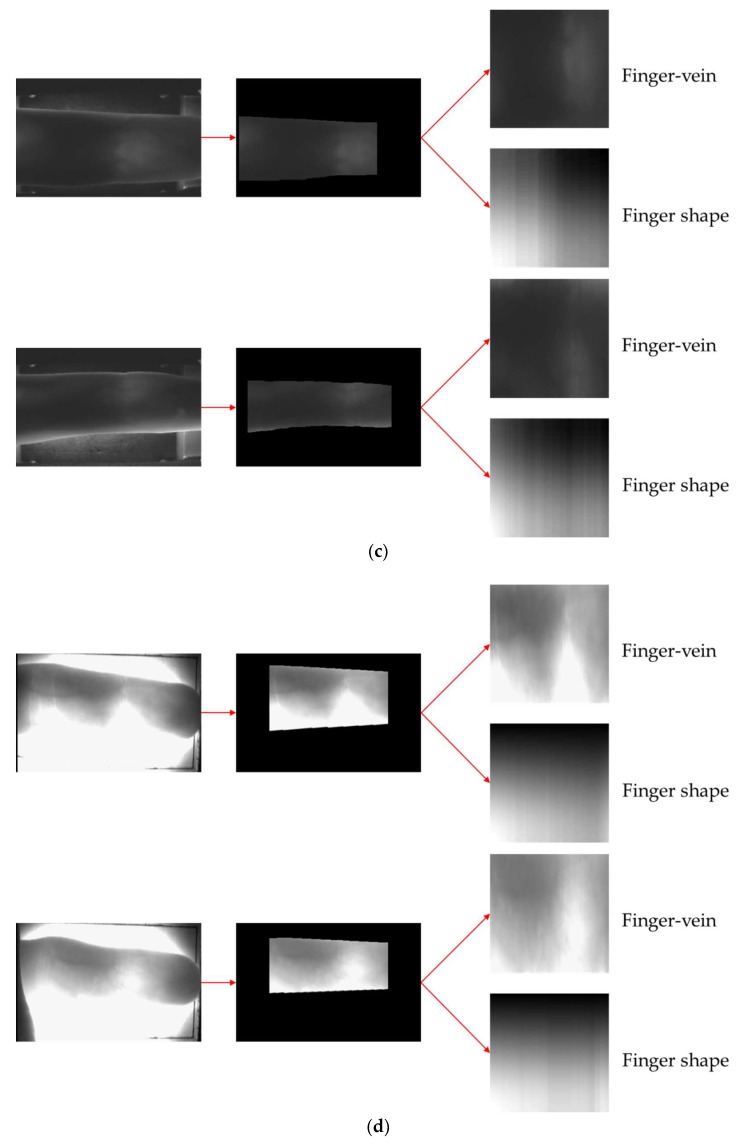
Examples of incorrect recognition cases. (**a**,**b**) False rejection cases with SDU-DB and PolyU-DB, respectively. (**c**,**d**) False acceptance cases with SDU-DB and PolyU-DB, respectively. In (**a**–**d**), upper and lower images show the enrolled and input images, respectively. In (**a**–**d**), left and middle images present the input and corresponding finger ROI. The right-upper images show that for obtaining the difference image for finger-vein recognition whereas the right-lower images present the spectrogram images for finger shape recognition**.**

Figure 13 shows the correct recognition cases by our method. Figure 13a,b show the correct acceptance cases, and the input images are correctly recognized as genuine matching, although there exists the misalignment of finger-vein positions between the enrolled and input images. Figure 13c,d present the correct rejection cases, which means the enrolled and input images are from a different class. As shown in Figure 13c,d, input images are correctly rejected as imposter matching based on the difference of finger shapes between the enrolled and input images, although finger-vein patterns are difficult to be observed in these two images due to low illumination. Figure 14 shows the error cases of the method proposed in this study. False rejection cases mainly occur because of the misalignment between finger-vein images due to finger position changes between the enrolled and recognized images (Figure 14a,b), and because of the difference between finger shape spectrogram images due to finger rolling (Figure 14a,b). False acceptance cases mainly occur because areas of finger are so dark (Figure 14c) or bright (Figure 14d). In addition, in the case of Figure 14d, the correct boundary of finger shape cannot be extracted by the highly saturated region inside of finger. To solve these problems, the study on compensation method of severe finger rolling and illumination variation is necessary as the future work.

## 5. Conclusions

In this study, finger-vein and finger shape multimodal biometrics based on a deep convolutional neural network (CNN) were proposed. Following are the details about what we achieved, the scientific contributions, how much important, and from which point of view, our results are different from the state of the art.

First, convex polygons were generated using the algorithm that finds the coordinates of the outermost pixels of finger ROIs to calibrate the empty spaces of the images. Then, the robust finger ROIs for misalignment were extracted after conducting in-plane rotation compensation based on the angle of tilting measured based on the boundaries of the upper, lower, left, and right pixels.

Second, two-dimensional spectrogram images that express finger-thickness frequency-component changes depending on the horizontal position of fingers were obtained and used as CNN inputs for finger shape recognition.

Third, matching distances calculated based on the features of finger-vein and finger shape obtained using ResNet models were score fused using various fusion methods, such as the weight sum, weighted product, and perceptron.

Fourth, trained CNN models and algorithms developed in this study were open through [30], so that other researchers can use them in fair performance evaluations.

Through an experiment conducted using two open databases, the accuracy of the method proposed in this study was confirmed to be higher than the state of the art, and those of existing methods and other CNN models. The experimental results revealed that most false rejection cases occurred because of misalignment between finger-vein images due to finger position changes between the enrolled and recognized images, and because of the difference between finger shape spectrogram images due to finger rolling. False acceptance cases occurred because the regions of finger were so dark or bright, and the correct boundary of finger shape could not be extracted by the highly saturated region inside of finger.

To solve these problems, the study on compensation method of severe finger rolling and illumination variation is necessary as the future work. Moreover, in future study, the possibility of performance improvement by combining the multimodal recognition method proposed in this study with scattering blur-restoration methods to reduce the blurring effects in finger-vein images shall be investigated.

## Figures and Tables

**Table 1:** Comparisons of proposed and previous research on finger-based recognition.

Category		Methods	Advantage	Disadvantage
Single modal based	Fingerprint	SVM-based quality estimation [1] and minutiae triplets [2]	Cost and size of system are most effective	- Vulnerable to fake attack - Affected by the skin condition of finger
	Finger knuckle print	Subspace [4], Local and global feature [5], Riesz transform [6] and Band-Limited Phase-Only Correlation (BLPOC) [5,9,10,31]	Less affected by the skin condition of finger than fingerprint recognition	More vulnerable to finger movement and skin deformations than fingerprint recognition
	Finger-vein	Radon transform [11], mean curvature [12], maximum curvature [13], Gabor filter [18,20], Hessian filter [19], fuzzy system [21], and convolutional neural network (CNN) [32,33]	- More resistant to fake attacks than fingerprint and finger knuckle-print recognition - Not affected by the skin condition of finger	Affected by shadows caused by NIR light, finger misalignment, and skin light scattering blur
	Finger shape	Wavelet transform [22], and Fourier descriptor and principal component analysis [23]	Not affected by the skin condition of finger	- Affected by thickness of finger according to age or health condition - The device size is bigger than fingerprint, finger knuckle-print, and finger-vein recognition devices - Extraction is hindered by stuck fingers
Multi-modal based	Multiple sensors based	Fusion of fingerprint and finger-vein [24], Fusion of finger-vein, finger shape, fingerprint, and finger knuckle print [25], fusion of finger-vein, fingerprint, and finger knuckle print [26], and fusion of finger-vein and finger knuckle print [27]	Better recognition performance than single-model methods by using 2 or more biometric traits	- High cost and large system size due to the use of 2 or more image-acquisition devices - Slow image-acquisition speed due to inability to acquire multimodal images simultaneously
	Single-sensor based	Handcrafted features and SVM [28,29]	Simultaneous finger-vein, fingerprint, and finger shape recognition using 1 device [28] Simultaneous finger-vein and finger shape recognition using 1 device [29]	Limited recognition performance improvement due to the use of handcraft features
		Deep features by CNN (proposed method)	- Simultaneous finger-vein and finger shape re cognition with 1 device - High recognition performance through the use of deep features	Requires intensive CNN training

**Table 2:** The proposed CNN configuration used in our research (3 * indicates that three pixels are included as padding in, respectively, the left, right, up, and down positions of an input image of 224 × 224 × 3 pixels, whereas 1 * means that one pixel is included as padding in the left, right, up, and down positions of the feature map) (2/1 ** means 2 at the first iteration and 1 from the second iteration) (in finger-vein recognition, #class is 2, and in finger shape recognition, it indicates the class number of the learning data).

Layer Name		Number of Filters	Size of Feature Map	Size of Filters	Number of Strides	Number of Padding	Number of Iterations
Image input layer			224 × 224 × 3				
Conv1		64	112 × 112 × 64	7 × 7 × 3	2	3 *	1
Max pool		1	56 × 56 × 64	3 × 3	2	0	1
Conv2	Conv2_1	64	56 × 56 × 64	1 × 1 × 64	1	0	3
	Conv2_2	64	56 × 56 × 64	3 × 3 × 64	1	1 *	
	Conv2_3	256	56 × 56 × 256	1 × 1 × 64	1	0	
	Conv2_4 (Shortcut)	256	56 × 56 × 256	1 × 1 × 64	1	0	
Conv3	Conv3_1	128	28 × 28 × 128	1 × 1 × 256	2/1 **	0	4
	Conv3_2	128	28 × 28 × 128	3 × 3 × 128	1	1 *	
	Conv3_3	512	28 × 28 × 512	1 × 1 × 128	1	0	
	Conv3_4 (Shortcut)	512	28 × 28 × 512	1 × 1 × 256	2	0	
Conv4	Conv4_1	256	14 × 14 × 256	1 × 1 × 512	2/1 **	0	23
	Conv4_2	256	14 × 14 × 256	3 × 3 × 256	1	1*	
	Conv4_3	1024	14 × 14 × 1024	1 × 1 × 256	1	0	
	Conv4_4 (Shortcut)	1024	14 × 14 × 1024	1 × 1 × 512	2	0	
Conv5	Conv5_1	512	7 × 7 × 512	1 × 1 × 1024	2/1 **	0	3
	Conv5_2	512	7 × 7 × 512	3 × 3 × 512	1	1*	
	Conv5_3	2048	7 × 7 × 2048	1 × 1 × 512	1	0	
	Conv5_4 (Shortcut)	2048	7 × 7 × 2048	1 × 1 × 1024	2	0	
AVG pool		1	1 × 1 × 2048	7 × 7	1	0	1
FC layer			#class				1
Softmax			#class				1

**Table 3:** Descriptions of two databases used in our research.

			SDU-DB	PolyU-DB
Original images		# of images	3816	1872
		# of people	106	156
		# of hands	2	1
		# of fingers	3 (index, middle, and ring fingers)	2 (index and middle fingers)
		# of classes (# of images per class)	636 (6)	312 (6)
Data augmentation for training	Finger shape image	# of images	230,868 (318 classes × 6 images × 121 times)	113,256 (156 classes × 6 images × 121 times)
	Finger-vein image	# of images	48,972	24,024
		# of images for genuine matching	24,486 (6 images × (13 times − 1) × 318 classes)	12,012 (6 images × (13 times − 1) × 156 classes)
		# of images for imposter matching (Random selection)	24,486	12,012

**Table 4:** Descriptions of initial parameters for training of various CNN models (“# of” represents “the number of”).

Dataset		# of Output Class	CNN Model	Max. # of Iteration (Epoch)	Mini-Batch Size	Learning Rate	Momentum/Gamma
SDU-DB	Finger-vein	2	VGG Net-16	7651 (10)	64	0.001	0.9/0.1
			ResNet-50	24,486 (10)	20		
			ResNet-101	32,648 (10)	15		
	Finger shape	318	VGG Net-16	36,073 (10)	64		
			ResNet-50	115,434 (10)	20		
			ResNet-101	153,912 (10)	15		
PolyU-DB	Finger-vein	2	VGG Net-16	3753 (10)	64		
			ResNet-50	12,012 (10)	20		
			ResNet-101	16,016 (10)	15		
	Finger shape	156	VGG Net-16	17696 (10)	64		
			ResNet-50	56,628 (10)	20		
			ResNet-101	75,504 (10)	15		

**Table 5:** Comparison of finger-vein recognition accuracy (unit: %) (The “number*” is referred from [19]).

Method		EER	
		SDU-DB	PolyU-DB
Non-training based method	Maximum Curvature [13]	4.54*	3.51
	Repeated line tracking [15]	5.46*	2.17
	Wide line detector [16]	22.7*	1.80
	Gabor + LBP [18]	8.096	3.61
Training-based method	VGG Net-16 [32]	3.906	2.491
	ResNet-50	3.4931	1.3435
	ResNet-101	3.3653	1.0779
	Score-level fusion of ResNet-50 and ResNet-101 by weighted product rule	3.0653	0.8888
Non-training and training-based method	Score-level fusion of Gabor + LBP [18] and ResNet-101 by weighted product rule	3.2426	0.9138

**Table 6:** Comparison of finger shape recognition accuracy (unit: %) (*: ResNet-50 is used for SDU-DB whereas ResNet-101 is used for PolyU-DB).

Method		EER	
		SDU-DB	PolyU-DB
Non-training-based method	Fourier descriptor (FD) [29]	13.8753	22.855
	FD + PCA [28]	13.574	22.8718
Training-based method	VGG Net-16 [56]	13.2758	19.2294
	ResNet-50	7.98	10.914
	ResNet-101	8.305	9.9553
	Score-level fusion of ResNet-50 and ResNet-101 by weighted product rule	7.5665	9.5631
Non-training and training-based method	Score-level fusion of FD + PCA [28] and ResNet * by weighted product rule	7.8147	9.8094

**Table 7:** Comparison of the accuracies of finger shape recognition according to one channel and three channel spectrogram images (unit: %).

Method		EER	
		SDU-DB	PolyU-DB
1 channel gray spectrogram image (Proposed method)	1st fold	5.35	10.096
	2nd fold	11.26	9.8146
	Average	8.305	9.9553
3 channels color spectrogram image	1st fold	6.431	13.748
	2nd fold	14.711	18
	Average	10.571	15.874

**Table 8:** Comparison of the accuracy of multimodal recognition according to various score-level fusion methods (unit: %).

Method		EER					
		SDU-DB			PolyU-DB		
Model		VGG Net-16	ResNet-50	ResNet-101	VGG Net-16	ResNet-50	ResNet-101
Score-level fusion	Weighted sum	3.892	2.4718	2.4258	2.491	1.1145	0.8255
	Weighted product	3.7217	2.4015	2.4088	2.3763	1.143	0.8265
	Perceptron	3.8516	2.3445	2.398	2.4433	1.0235	0.7859
	Bayesian	4.3621	4.3857	3.509	3.7196	1.0965	1.706
